# Modulation of SERCA1a by Sesquiterpene Lactones: Insights into Inhibition Mechanisms

**DOI:** 10.1007/s12013-026-02097-x

**Published:** 2026-06-10

**Authors:** Petronela Rezbáriková, Jana Viskupičová, Magdaléna Májeková, Silvia Micháliková, Michal Jurášek, Pavel Drašar, Juraj Harmatha, Eva Kmoníčková

**Affiliations:** 1 Centre of Experimental Medicine, Institute of Experimental Pharmacology and Toxicology, Slovak Academy of Sciences, Bratislava, Slovak Republic; 2 Department of Chemistry of Natural Compounds, University of Chemistry and Technology, Prague, Czech Republic; 3 Institute of Organic Chemistry and Biochemistry, Czech Academy of Sciences, Prague, Czech Republic; 4 Department of Pharmacology, Second Faculty of Medicine, Charles University, Prague, Czech Republic

**Keywords:** Ca²⁺-ATPase, sesquiterpene lactones, thapsigargin, kinetic parameters, molecular modeling

## Abstract

The sarcoplasmic reticulum Ca²⁺-ATPase (SERCA1a) is a key regulator of intracellular calcium homeostasis and an emerging therapeutic target in cancer, cardiac, and metabolic diseases. Sesquiterpene lactones such as thapsigargin (TG) and trilobolide (TB) inhibit SERCA1a via transmembrane binding, yet systematic comparisons of their inhibitory potency and biophysical mechanisms remain limited. Here, we investigated the inhibitory effects of TG, TB, and two TB derivatives (TB-44, TB-46(2)) using kinetic, thermodynamic, and docking analyses. SERCA1a activity in isolated sarcoplasmic reticulum vesicles was quantified by an NADH-coupled ATPase assay, with Ca²⁺- and ATP-dependence modeled by Michaelis–Menten and Hill fits. All compounds inhibited SERCA1a in a dose-dependent manner, with TB most potent (IC₅₀ = 0.36 µM vs. TG 0.60 µM) and TB-44 the most effective derivative (IC₅₀ = 1.47 µM). Each reduced V_max_ while largely preserving Ca²⁺ sensitivity, consistent with stabilization of a low-turnover SERCA1a conformation and impaired ATP-driven catalysis. Notably, TB’s enhanced potency, despite intermediate calculated binding energy (ΔG = − 9.1 kcal/mol), correlated with moderate lipophilicity (logP = 2.43) and favorable electrostatic contacts with Gln259. These data highlight that effective SERCA1a inhibition by sesquiterpene lactones reflects an interplay between lipophilicity, membrane partitioning, and electrostatic–hydrophobic complementarity, rather than binding affinity alone.

## Introduction

Calcium signaling orchestrates essential processes in nearly all eukaryotic cells, including muscle contraction, neural signaling, metabolism, and cell death [1, 2]. Disrupted calcium homeostasis is implicated in diverse pathologies, including heart failure [3], neurodegeneration [4], cancer [5], metabolic disorders [6] and immunodeficiency [7], highlighting the central role of precise calcium regulation in cellular and organismal health.

The sarcoplasmic reticulum Ca²⁺-ATPase (SERCA) plays a pivotal role in actively transporting Ca²⁺ ions from the cytosol into the sarcoplasmic reticulum (SR) lumen, thereby maintaining intracellular calcium balance. Structurally, SERCA comprises three cytoplasmic domains – the actuator (A), nucleotide-binding (N), and phosphorylation (P) domains – and a transmembrane domain formed by 10 helices that coordinate ion transport [8]. Its function is driven by an E1/E2 conformational cycle involving sequential Ca²⁺ binding, ATP phosphorylation, and the release of ADP and inorganic phosphate [9]. Given SERCA’s central role in maintaining calcium homeostasis, even subtle disruptions in its conformational dynamics can have profound physiological consequences.

SERCA1a is the best-characterized isoform of the SERCA family, predominantly expressed in fast-twitch skeletal muscle fibers [10]. Extensive structural and functional studies have elucidated its catalytic cycle, conformational transitions, and regulatory mechanisms, establishing SERCA1a as a robust model for mechanistic studies and drug screening [11]. SERCA1a is highly conserved across species, with homologous orthologs in other mammals sharing strong sequence and structural similarity, which further supports its use as a model in comparative and translational studies [12, 13]. Understanding the structure-activity relationships of SERCA1a inhibitors is critical for developing selective modulators with therapeutic potential in cancer, cardiac disease, and metabolic disorders.

Sesquiterpene lactones (STLs) are a diverse group of naturally occurring terpenoids with a core 15-carbon skeleton [14]. STLs have gained attention for their broad biological properties, including anti-inflammatory [15], anticancer [16, 17], antioxidant [18], and antimicrobial effects [19]. These activities are primarily attributed to the presence of α,β-unsaturated γ-lactone rings, which confer high reactivity toward nucleophilic sites on cellular proteins [20]. Among STLs, SERCA1a inhibitors have emerged as promising tools for both mechanistic studies and potential therapeutics.

Thapsigargin (TG) is a potent STL that binds with high affinity to a hydrophobic pocket within the transmembrane domain of SERCA1a, stabilizing the enzyme in its inactive E2 conformation and preventing Ca²⁺ binding. This mechanism disrupts calcium homeostasis by elevating cytosolic Ca²⁺ levels [14]. Trilobolide (TB), a structurally related compound, was hypothesized to interact with SERCA1a similarly based on structural homology to TG [21].

Novel derivatives of trilobolide (Fig. 3) have been synthesized to probe intracellular behavior and bioactivity.

**Fig. 1:**
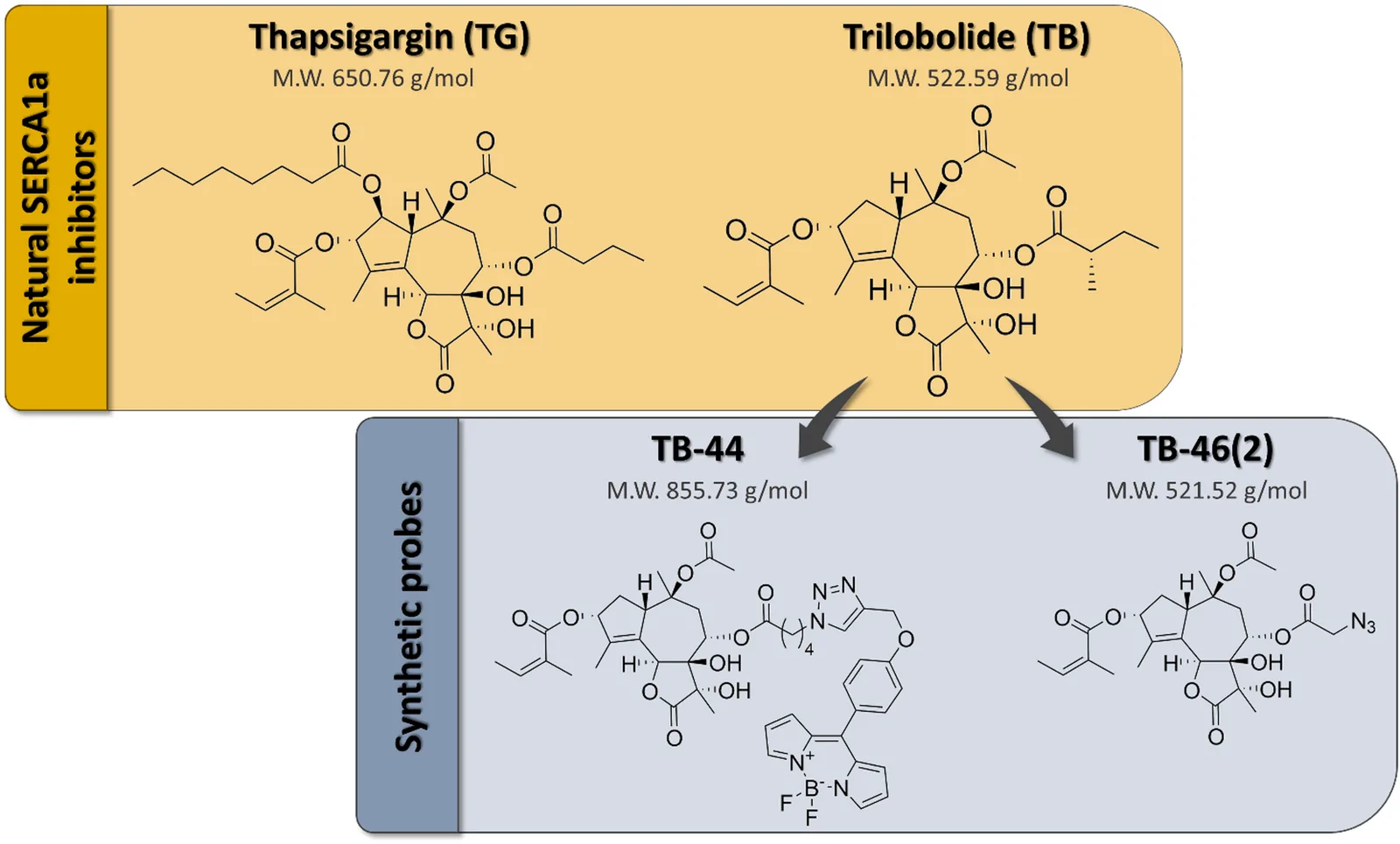
Chemical structures of natural SERCA1a inhibitors (thapsigargin and trilobolide) and their synthetic derivatives. TB-44 is a fluorescent BODIPY-conjugate probe, and TB-46(2) is an azide-modified analog for click-chemistry applications. Molecular weights are indicated for each compound. Detailed synthetic procedures and compound characterization were reported previously [21]

In particular, TB-44 was developed as a fluorescent conjugate and was shown to penetrate cells and localize within the endoplasmic reticulum (ER), similarly to its parent compound [21]. Because SERCA1a is a major ER/SR membrane-targeted functional protein, structural modifications that affect membrane partitioning or binding-site accessibility may directly influence inhibitory potency.

Despite TG’s role as a “model” SERCA1a inhibitor, a systematic side-by-side comparison of thapsigargin, trilobolide, and synthetic TB derivatives linking in vitro potency, kinetic mechanisms, and molecular docking remains lacking. Insight into the molecular determinants and energetics of their interactions is essential for advancing SERCA1a-targeted therapeutics.

Here, we combined enzyme kinetics, thermodynamic and structure-based molecular docking to:

1. Quantify the effects of TG, TB, TB-44, and TB-46(2) on Ca²⁺- and ATP-dependent SERCA1a inhibition.
2. Elucidate how structural modifications and lipophilicity influence inhibitory potency and mechanism.
3. Correlate experimental inhibition profiles with calculated binding affinities and predicted molecular interactions.

These findings reveal how lipophilicity and transmembrane interactions determine the potency of sesquiterpene lactones against SERCA1a, guiding future inhibitor optimization.

## Materials and Methods

### Chemicals

Unless otherwise indicated, all chemicals were at least reagent grade and purchased from Merck/ MilliporeSigma (DE). Thapsigargin was purchased from Merck (DE).

New trilobolide derivatives were synthesized via selective structural modifications at the C-3 position of the sesquiterpene lactone scaffold. The synthetic route involved derivatization of the hydroxy group through esterification or carbamate formation using various acyl or alkyl isocyanate reagents. The resulting compounds were purified by chromatographic techniques and structurally confirmed by NMR and mass spectrometry [22].

A fluorescent trilobolide derivative was synthesized by selectively modifying the C-8 hydroxyl group of trilobolide to introduce a linker bearing an azide moiety. This intermediate was subsequently conjugated with a BODIPY fluorophore via a copper-catalyzed azide-alkyne cycloaddition. The final compound was purified by chromatographic techniques and characterized by NMR and mass spectrometry, as reported previously in the Supplementary Information [21].

### Isolation of SR

Sarcoplasmic reticulum (SR) vesicles were isolated from the spinal region of the fast-twitch skeletal muscle and the hind limbs of a female New Zealand rabbit (about 2.5 kg), according to the protocol of Warren et al. [23, 24], with modifications by Andriamainty et al. [25]. The isolated skeletal SR vesicles contain approximately 80% SERCA1a, with the remainder comprising calsequestrin [26, 27].

### Ca^2+^-ATPase Activity Measurement

Sarcoplasmic reticulum Ca^2+^-ATPase (SERCA1a) activity was measured spectrophotometrically by a NADH-coupled enzyme assay, as outlined by Warren et al. [23, 24]. Stock solutions of test compounds (10 mM) were prepared in DMSO and aliquots were stored at -20 °C. The assay mixture containing Hepes (40 mM, pH 7.2), KCl (0.1 M), MgSO_4_ (5.1 mM), ATP (2.1 mM), phosphoenolpyruvate (0.52 mM), EGTA (1 mM), NADH (0.15 mM), pyruvate kinase (7.5 IU), lactate dehydrogenase (18 IU), and A23187 calcium ionophore (1 µM) was incubated at 37 °C for 2 min. The reaction mixture comprised 100 µg/ml SERCA1a and compounds at their respective concentrations. The final amount of SR vesicles was 12.5 µg protein/cuvette. The reaction was initiated by the addition of 1 mM CaCl_2_. The rate of Ca²⁺-dependent ATP hydrolysis was monitored by measuring the decrease in NADH absorbance at 340 nm using a SPECORD 40 spectrophotometer (Analytik Jena, DE) at 37 °C.

### Kinetic Parameters of Ca^2+^-ATPase Activity

Two independent experimental sets were conducted using SR preparations isolated from different animals on separate days. Set 1 comprised thapsigargin (TG) and TB-44 treatments; Set 2 comprised trilobolide (TB) and TB-462 treatments. Each set included its own matched control, tested on the same day to account for biological variability between independent SR preparations. All kinetic parameters and derived values (pATP, pCa, ΔΔG, Hill coefficients) were calculated relative to the respective same-day control within each set. This within-set normalization approach ensures valid thermodynamic calculations; direct cross-set comparisons of absolute kinetic values are therefore not appropriate.

#### ATP Dependence

ATP dependence of SERCA1a was measured using the NADH-coupled enzyme assay as described above. Samples were pre-incubated with SR vesicles and the assay mixture (without ATP) at 37 °C and pH 7.2. Pre-warmed ATP (at 37 °C in 40 mM Hepes, pH 7.2) was then added in final concentrations ranging from 0.1 µM to 2.1 mM. 1 mM CaCl₂ initiated the reaction. The dependence of Ca^2+^-ATPase activity on ATP was modeled using a Michaelis-Menten equation:

$${\mathrm{Activity}} = \frac{{\rm{V}}^{'}_{\rm{max}} \cdot [{\rm Mg.ATP}]}{{\rm K}^{ '}_{\rm m}+ [{\rm Mg.ATP}]}$$

where ${\rm{V}}_{\rm{max}}^{'}$ is the activity at saturating concentrations of the substrate and ${\rm{K}}_{\mathrm{m}}^{'}$ is the Michaelis constant. ATP affinity was also expressed as pATP₅₀ = 3 − log₁₀(K_m_ [mM]), and thermodynamic changes were calculated as ΔΔG = − 5.93 × ΔpATP₅₀ (kJ·mol⁻¹ at 37 °C). All compound treatments were compared to same-batch, same-day controls to account for batch-to-batch variability.

#### Ca^2+^ Dependence

Ca²⁺ dependence of SERCA1a activity (0.024 – 13.5 µM range) was assessed by pre-incubating reconstituted vesicles with 2.1 mM ATP at 37 °C and pH 7.2 and initiating reactions with Ca²⁺. Free Ca^2+^ concentrations ([Ca^2+^]_free_) were calculated using the Maxchelator program and based on binding affinities described by Gould et al. [28]. Ca²⁺-activation curves were fit with the Hill equation to obtain V_max_, K_0.5_, and the Hill coefficient h:

$${\rm Activity} = {\rm V}_{\rm max} \times [{\rm Ca}^{2+}]^{\rm h} / {{\rm K}_{0.5}}^{\rm h} + [{\rm Ca}^{2+}]^{\rm h}$$

where V_max_ is the activity of Ca^2+^-ATPase at saturating substrate concentrations, K_0.5_ is the Ca^2+^ concentration corresponding to half-maximal activity, and h is a phenomenological descriptor of curve steepness under our assay conditions (not interpreted as the number of binding sites). All compound treatments were compared to same-batch, same-day controls to account for batch-to-batch variability in reconstituted preparations. The Ca²⁺ affinity was also expressed as pCa₅₀ = 6 − log₁₀(K₀.₅ [µM]), and thermodynamic changes were calculated as ΔΔG = − 5.93 × ΔpCa₅₀ (kJ·mol⁻¹ at 37 °C).

### In Silico Study

The structures of the test compounds were obtained using Spartan’20 software (Wavefunction, Inc., CA, USA, version 1.1.4) [29], with the geometry of the azulene ring derived from thapsigargin and optimized using the MMF94 force field. Molecular docking was performed with the Molecular Operating Environment (Chemical Computing Group ULC, QC, Canada, version 2019.01) [30].

The docking process used the SERCA1a crystal structure (PDB code 2ear, stabilized with thapsigargin), prepared using the QuickPrep protocol for protein structure preparation and protonation. We employed a triangle matcher, the London dG score for basic docking, and the GBVI/WSA ΔG score for induced-fit geometry refinement. Ligands and proteins were protonated at a physiological pH. The docking site was defined as the space within 5 Å of the thapsigargin surface in the X-ray position. Binding affinities were expressed as GBVI/WSA ΔG score functions. Additionally, the potential interaction energies ΔE (INT in MOE) and their electrostatic (ΔE_elst_) and van der Waals (ΔE_vdw_) components were calculated after the complete optimization of each complex. The MOE program also calculated the partition coefficient (logP) and distribution coefficient (logD7.4).

### Statistical Evaluation

Statistical evaluation was performed using one-way ANOVA followed by Dunnett’s multiple comparisons test. Data are presented as mean ± SD/SEM (as specified in each figure legend) of at least three independent experiments, with each sample measured in duplicate or more. Statistical significance was set at * *p* < 0.05, ** *p* < 0.01, *** *p* < 0.001. All kinetic fits, statistical tests, and graph generation were performed using GraphPad Prism (GraphPad Software, CA, USA, version 8).

## Results

### Effect of Sesquiterpene Lactones on SERCA1a Activity

**Fig. 2:**
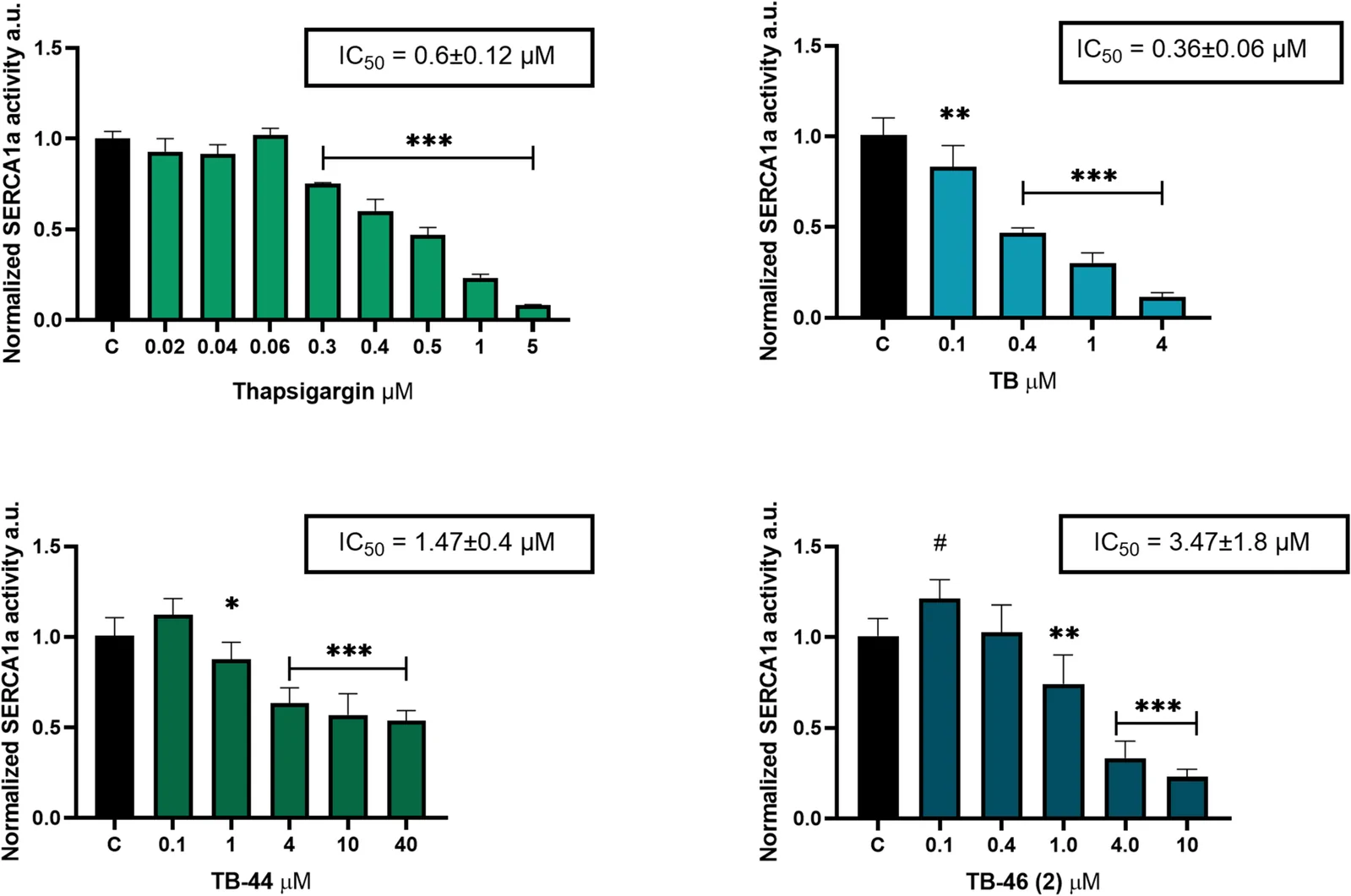
The impact of tested compounds on SERCA1a activity. SR vesicles (0.1 mg/ml protein) were pre-incubated for 2 min at 37 °C and pH 7.2 with individual concentrations of sesquiterpene lactones and their derivatives. The enzyme activity in the presence of sesquiterpene compounds was monitored continuously for 3 min. Values are mean ± SD of three independent experiments, where each sample was measured in two parallel experiments. ♯ *p* < 0.05, * *p* < 0.05, ** *p* < 0.01, *** *p* < 0.001 indicate a significant difference between the control sample and the treated samples

We investigated the effects of thapsigargin (TG), trilobolide (TB), and two trilobolide derivatives (TB-44 and TB-46(2)) on the Ca^2+^-ATPase activity of SERCA1a, with particular attention to kinetic parameters related to ATP and Ca^2+^ binding. Under optimal assay conditions (2.1 mM ATP, 13.5 µM CaCl₂), all tested compounds significantly inhibited SERCA1a activity in a dose-dependent manner (Fig. 2). As expected, TG demonstrated potent inhibitory activity at low concentrations, with an IC_50_ of 0.6 ± 0.12 µM, consistent with its well-established potency [31]. Interestingly, trilobolide (TB) emerged as the most potent inhibitor in this study, with an IC_50_ of 0.36 ± 0.06 µM, suggesting higher inhibitory efficiency than TG under the same experimental conditions. The TB derivatives were evaluated over a wide range of concentrations. TB-44 inhibited SERCA1a activity between 1 and 40 µM, with an IC_50_ of 1.47 ± 0.4 µM and TB-46(2) showed inhibition in the 1–10 µM range, yielding an IC_50_ of 3.47 ± 1.8 µM. These results indicate that structural modifications to the trilobolide scaffold significantly attenuate SERCA1a inhibition, with TB-46(2) exhibiting approximately 10-fold lower potency compared to the parent compound trilobolide.

### Effects of Sesquiterpene Lactones and Their Derivatives on ATP-Dependent Kinetics and Thermodynamics

To elucidate the mechanism of SERCA1a inhibition by sesquiterpene lactones, detailed kinetic analyses were performed at concentrations approximating their respective IC₅₀ values: TG (0.5 µM; IC₅₀ = 0.6 µM), TB (0.4 µM; IC₅₀ = 0.36 µM), TB-44 (4 µM; IC₅₀ = 1.47 µM), and TB-46(2) (4 µM; IC₅₀ = 3.47 µM). This experimental design allows for a direct comparison of inhibitory mechanisms under conditions where each compound produces approximately 50% enzyme inhibition, enabling assessment of both substrate binding affinity (K_m_ or K₀.₅) and changes in maximal catalytic capacity (V_max_). At these concentrations, the effects on both competitive and non-competitive components of inhibition can be reliably distinguished through Michaelis-Menten and Hill analyses, providing insight into whether tested compounds primarily affect substrate binding, catalytic efficiency, or both.

**Fig. 3:**
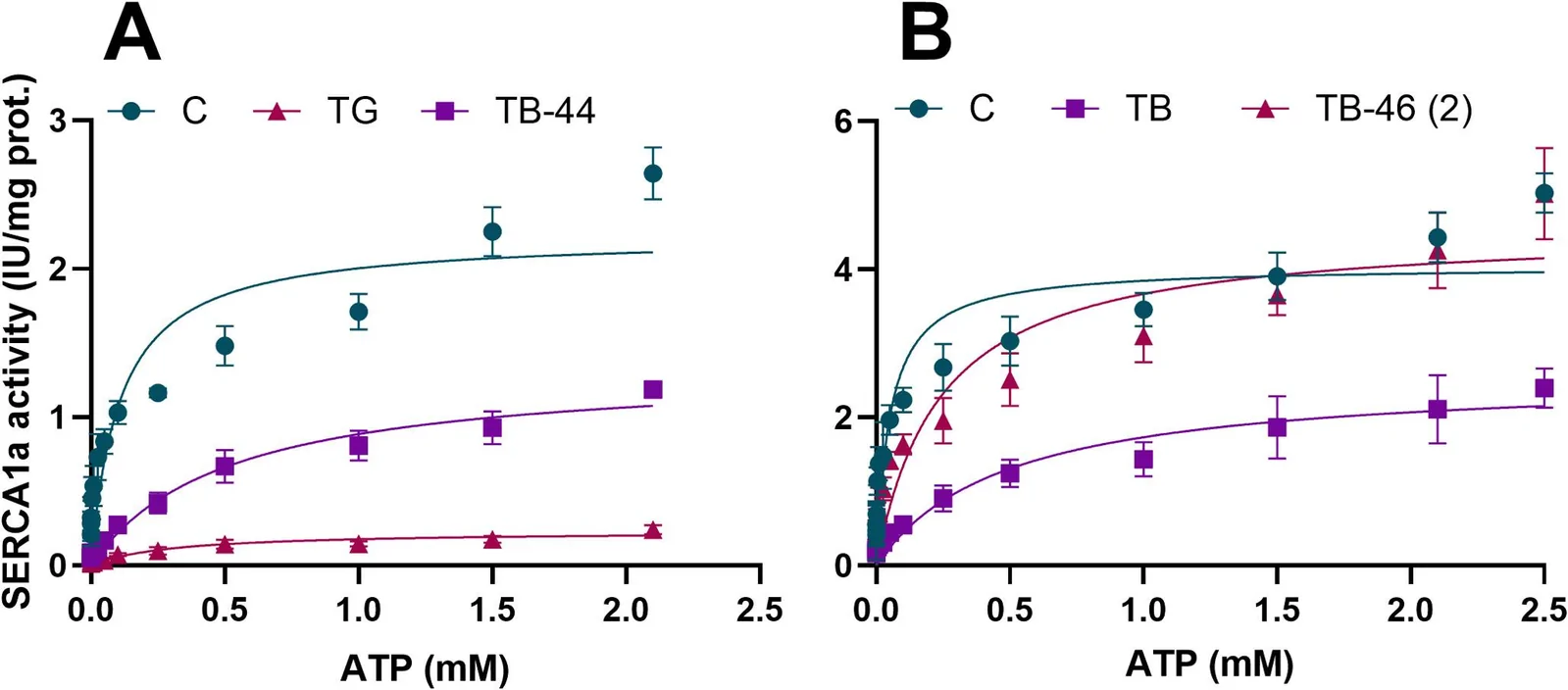
ATP-dependence of SERCA1a activity in the presence of sesquiterpene lactones and derivatives: activity in the presence of **(A)** control (C), thapsigargin (TG, 0.5 µM) and TB-44 (4 µM); **(B)** control (C), trilobolide (TB, 0.4 µM) and TB-46(2) (4 µM). SERCA1 activity was determined as mentioned in Materials and Methods. Solid lines are Michaelis–Menten fits. Symbols show mean ± SD from *n* = 4 independent experiments; error bars are plotted where SD exceeds the symbol size (otherwise not visible). Graphs, fits, and related parameters were created/calculated using GraphPad Prism 8.0.1 (GraphPad Software, CA, USA)

In the presence of TG (0.5 µM) and TB-44 (4 µM), substantial alterations in both maximal reaction rate and ATP binding affinity were observed (Fig. 3A ). For the TG/TB-44 experimental set, control samples exhibited a V_max_ of 2.22 ± 0.01 IU/mg and a K_m_ of 0.11 ± 0.002 mM (pATP₅₀ = 3.96). Thapsigargin treatment resulted in a significant reduction of V_max_ to 0.23 ± 0.01 IU/mg (representing only 10% of the control V_max_) and an increase in K_m_ to 0.304 ± 0.063 mM. TB-44 treatment reduced V_max_ to 1.33 ± 0.054 IU/mg (60% of control) with K_m_ increasing to 0.51 ± 0.059 mM. Analysis of pATP₅₀ values shows that ATP affinity declines in the presence of compounds (Fig. 3A). However, this reduction is less pronounced than the loss in catalytic efficiency (V_max_/K_m_), indicating that the major kinetic deficit results from impaired catalytic turnover rather than substrate binding alone. Catalytic efficiency (V_max_/K_m_) was markedly reduced by both treatments, with TG retaining only 3.7% and TB-44 12.9% of the control value (Table 1). The ΔΔG values, calculated from experimental kinetic measurements, represent thermodynamic changes in ATP binding affinity - measuring how the inhibitor shifts the enzyme’s ability to bind its natural substrates. Positive ΔΔG values indicate that the tested compounds decrease substrate binding affinity (a less favorable binding equilibrium), which directly correlates with reduced catalytic efficiency. Overall, treatment with TG and TB-44 substantially impairs ATP-dependent Ca^2+^-ATPase activity primarily through reductions in maximal reaction rate and catalytic efficiency, highlighting functional deficits induced by treatment.

The second group contained TB and its derivative, TB-46(2), which underwent the same treatment (Fig. 3B). TB (0.4 µM) substantially diminished the SERCA1a ATP-dependent V_max_ to 2.6 ± 0.13 IU/mg while markedly increasing K_m_ to 0.48 ± 0.077 mM in comparison to control (V_max_ 4.04 ± 0.12 IU/mg, K_m_ to 0.052 ± 0.009 mM). TB-46(2) (4 µM) exhibited milder inhibitory effects, maintaining V_max_ at 4.55 ± 0.18 IU/mg, while we observed an increase in K_m_ to 0.24 ± 0.04 mM (Fig. 3B).

Catalytic efficiency was reduced in both cases – TB to 6.96% of control and TB-46(2) to 24.0% of control (Table 1). The corresponding midpoint values (pATP₅₀ = 3.3 for TB and 3.6 for TB-46(2)) and ΔΔG values (+ 5.73 kJ·mol⁻¹ and + 3.98 kJ·mol⁻¹) indicated a more energetically unfavorable ATP-dependent activation in the inhibited groups. Although ATP affinity decreased, this change was less pronounced than the decline in catalytic efficiency, suggesting that the principal inhibition arises from impaired catalytic capacity (reduced V_max_) rather than reduced substrate binding.

**Table 1:** ATP-dependent kinetic parameters of SERCA1a in the presence of thapsigargin (TG), trilobolide (TB), and TB-derived chemical biology probes

		Kinetic parameters				Binding affinity		ΔΔG (kJ·mol⁻¹)	r^2^
		V_max_ (IU/mg)	K_m_ (mM)	V_max_/K_m_ (IU·mg⁻¹·mM⁻¹)	% of control efficiency	pATP₅₀	ΔpATP₅₀ vs. C		
**TG/TB-44 dataset**	**C**	2.22 ± 0.01	0.11 ± 0.002	20.40	100%	3.9	—	—	0.82
	**TG**	0.23 ± 0.01	0.304 ± 0.063	0.76	3.7%	3.5	−0.45	+ 2.64	0.82
	**TB-44**	1.33 ± 0.054	0.51 ± 0.059	2.61	12.8%	3.3	−0.67	+ 3.97	0.95
**TB/TB-46(2) dataset**	**C**	4.04 ± 0.12	0.052 ± 0.009	77.69	100%	4.3	—	—	0.83
	**TB**	2.60 ± 0.13	0.48 ± 0.077	5.41	6.96%	3.3	−0.97	+ 5.73	0.88
	**TB-46 (2)**	4.55 ± 0.18	0.24 ± 0.040	18.65	24%	3.6	−0.67	+ 3.98	0.84

Control (C) and treated samples from two independent experimental sets are shown: TG/TB-44 set and TB/TB-46(2) set, tested on different days using separate SR preparations, resulting in biological variability in baseline kinetic parameters. Parameters were obtained from Michaelis–Menten fits: pATP₅₀ = 3 − log₁₀(K_m_ [mM]); ΔΔG = − 5.93 × ΔpATP₅₀ (kJ/mol at 37 °C). “% of control efficiency” = (V_max_/K_m_)_drug_/(V_max_/K_m_)_C_ × 100%. Values are mean ± SEM from *n* = 4 independent experiments; r² indicates fit quality. Kinetic parameters V_max_ (ATPase activity at saturating ATP concentration) and K_m_ (Michaelis constant) were calculated from the fits. Values within each experimental set are directly comparable and normalized to their respective same-day control

### Effects of Sesquiterpene Lactones and Their Derivatives on Ca^2+^-Dependent Kinetics and Thermodynamics

To characterize the inhibitory mechanisms of thapsigargin-like compounds, we examined their effects on SERCA1a Ca²⁺-activation kinetics using Hill allosteric sigmoidal functions to determine key parameters including maximum velocity (V_max_), Ca²⁺ affinity (K₀.₅), Hill coefficient (h), and thermodynamic descriptors (pCa₅₀, ΔpCa₅₀, ΔΔG). The ΔΔG values for Ca²⁺-dependent kinetics, derived from Hill analysis of Ca²⁺-activation curves, represent thermodynamic changes in Ca²⁺ binding affinity – measuring how inhibitor binding affects the enzyme’s ability to recognize and bind calcium. Negative ΔΔG values indicate enhanced apparent Ca²⁺ affinity in the inhibited state, consistent with stabilization of Ca²⁺-bound conformations, whereas positive ΔΔG values indicate reduced Ca²⁺ affinity.

**Fig. 4:**
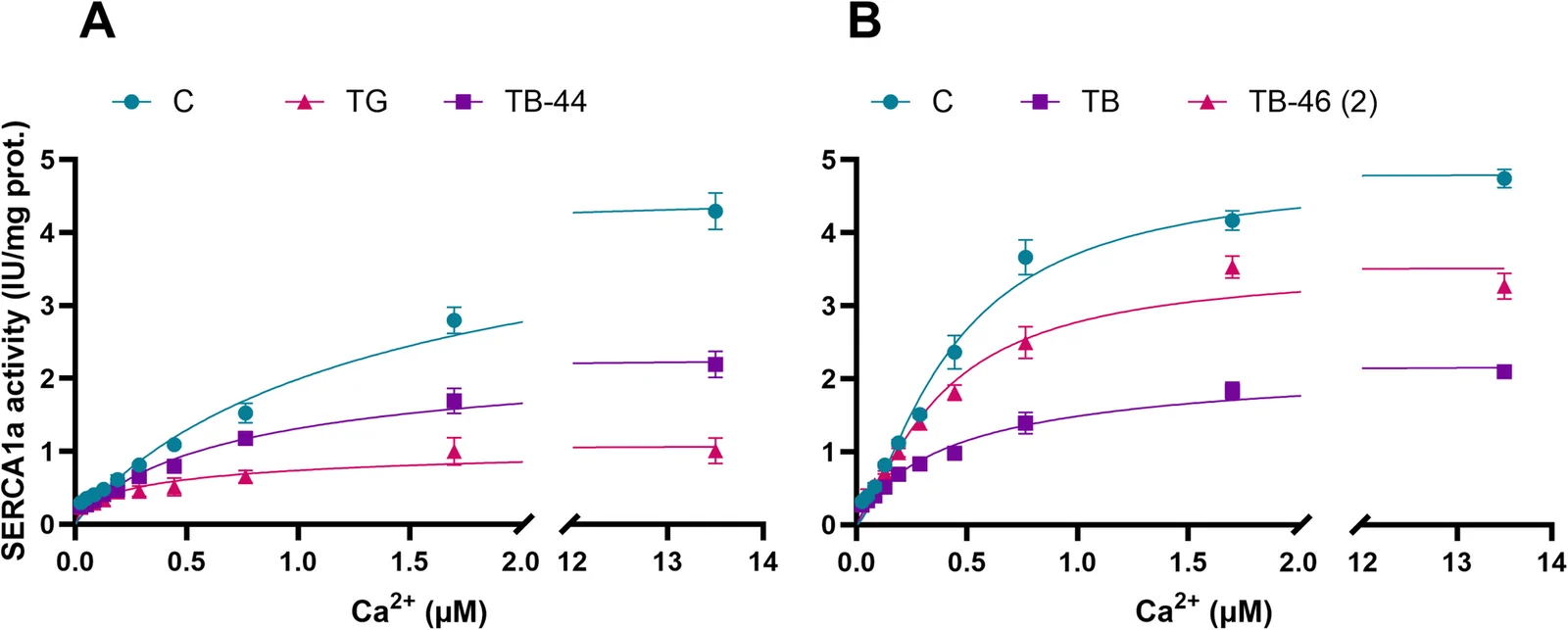
Ca^2+^-dependence of SERCA1a activity in the presence of sesquiterpene lactones and derivatives: activity in the presence of **(A)** control (C), thapsigargin (TG, 0.5 µM) and TB-44 (4 µM); **(B)** control (C), trilobolide (TB, 0.4 µM) and TB-46(2) (4 µM). SERCA1 activity was determined as mentioned in Materials and Methods. Solid lines are fitted by Hill (allosteric sigmoidal) function. Symbols show mean ± SD from *n* = 4 independent experiments; error bars are plotted where SD exceeds the symbol size (otherwise not visible). Graphs, fits, and related parameters were created/calculated using GraphPad Prism 8 software (GraphPad Software, San Diego, CA, USA)

The Ca²⁺-dependence of SERCA1a activity was characterized to assess both maximal catalytic capacity and Ca²⁺ sensitivity (Fig. 4). Concentrations of the tested compounds were identical to those used in ATP-dependent experiments, with compounds tested in two groups: TG with TB-44 (Fig. 4A), and TB with TB-46(2) (Fig. 4B).

In the TG and TB-44 group, the control V_max_ was higher (4.75 ± 0.087 IU/mg; K₀.₅ = 1.50 ± 0.15 µM). TG, as a known inhibitor, caused the most profound inhibition, lowering V_max_ to 21% of control (1.02 ± 0.046 IU/mg) and the Ca²⁺ affinity (K₀.₅) to 0.46 ± 0.17 µM. Changes in binding affinities of TG – positive ΔpCa₅₀ (+ 0.52) and a negative ΔΔG (-3.08 kJ·mol⁻¹) – are consistent with its known mechanism of stabilizing Ca²⁺-bound conformations (Table 2).

TB-44 reduced activity to 49% of control (2.31 ± 0.051 IU/mg), with K₀.₅ of 0.82 ± 0.09 µM (ΔpCa₅₀ = +0.27; ΔΔG = -1.6 kJ·mol⁻¹). Overall, TG and TB-44 significantly decreased V_max_ while Ca^2+^ sensitivity remained relatively stable despite changes in maximal activity. The Hill coefficient for control SERCA1a (h = 0.93 ± 0.04) was consistent with reported values for SERCA1a and other SERCA isoforms in reconstituted systems, which typically range from 0.9 to 2.1 depending on experimental conditions [32]. Hill coefficients decreased from 0.93 (control) to 0.62 (TG) and 0.87 (TB-44), indicating reduced cooperativity of Ca^2+^ activation.

In the presence of TB and TB-46(2), control samples exhibited a maximum V_max_ of 5.22 ± 0.12 IU/mg and a Ca²⁺ affinity (K₀.₅) of 0.43 ± 0.018 µM. The enzymatic activity in response to varying Ca^2+^ concentrations was significantly decreased in the presence of TB, resulting in a significant reduction of V_max_ to 42% of control (2.18 ± 0.041 IU/mg), with slight alterations in Ca²⁺ affinity (K₀.₅ = 0.48 ± 0.041 µM), and decreases in Hill coefficient (h = 0.88 ± 0.045). Ca²⁺ binding affinity was affected only moderately, as shown by a minor negative shift in ΔpCa₅₀ and a small positive ΔΔG. TB-46(2) preserved a greater proportion of activity (3.74 ± 0.097 IU/mg; 72% of control) with K₀.₅ of 0.37 ± 0.024 µM. Ca^2+^ affinity binding was not significantly affected as shown by minor changes in ΔpCa₅₀ shift (+ 0.06) and ΔΔG (-0.36 kJ·mol⁻¹). The Hill coefficient (1.31 ± 0.081) remained closer to control values, indicating preservation of cooperative Ca²⁺ binding (Table 2).

Across both datasets, the dominant effect of all tested compounds was a substantial reduction in V_max_, while Ca^2+^ sensitivity (K_0.5_/pCa_50_) showed only minor, statistically non-significant variations. The small ΔΔG values for Ca^2+^ binding ( ≤ ~ 3 kJ·mol^− 1^) contrast sharply with the large energetic penalties observed for ATP-dependent activation, indicating that these compounds primarily impair catalytic capacity rather than Ca^2+^ recognition. Reductions in Hill coefficients suggest some loss of cooperative Ca^2+^ binding, most pronounced with TG and TB.

**Table 2:** Ca²⁺-dependent kinetic parameters of SERCA1a in the presence of thapsigargin (TG), trilobolide (TB), and TB-derived chemical biology probes

		Kinetic parameters			Binding affinity		ΔΔG (kJ·mol⁻¹)	h	r^2^
		V_max_ (IU/mg)	K_0.5_ (µM)	% of control V_max_	pCa_50_	ΔpCa₅₀ vs. C			
TG/TB-44 dataset	C	4.75 ± 0.087	1.50 ± 0.15	100%	5.8	-	-	0.93 ± 0.04	0.98
	TG	1.02 ± 0.046	0.46 ± 0.17	21%	6.3	+ 0.52	-3.08	0.62 ± 0.29	0.81
	TB-44	2.31 ± 0.051	0.82 ± 0.09	49%	6.1	+ 0.27	-1.6	0.87 ± 0.049	0.96
TB/TB-46(2) dataset	C	5.22 ± 0.12	0.43 ± 0.018	100%	6.4	-	-	1.42 ± 0.062	0.98
	TB	2.18 ± 0.041	0.48 ± 0.041	42%	6.3	-0.05	+ 0.3	0.88 ± 0.045	0.97
	TB-46(2)	3.74 ± 0.097	0.37 ± 0.024	72%	6.4	+ 0.06	-0.36	1.31 ± 0.081	0.96

Control (C) and treated samples from two independent experimental sets are shown. TG/TB-44 set and TB/TB-46(2) set were tested on different days using separate SR preparations, resulting in biological variability in baseline kinetic parameters. Parameters were obtained by fitting Hill (allosteric sigmoidal) functions to activity versus [Ca²⁺]. pCa₅₀ = 6 − log₁₀(K₀.₅ [µM]); ΔΔG = − 5.93 × ΔpCa₅₀ (kJ/mol at 37 °C). Values are mean ± SEM from *n* = 4 independent experiments; *r²* indicates goodness of fit. *V*_*max*_ is the maximal ATPase rate under the assay conditions; *K*_*0.5*_ is the Ca²⁺ concentration giving half-maximal activity; *h* is the Hill slope. Values within each experimental set are directly comparable and normalized to their respective same-day control

### Docking and Analysis of Compound–SERCA1a Interactions

Molecular docking simulations were performed to investigate the binding modes and interaction patterns of thapsigargin (TG), trilobolide (TB), TB-44, and TB-46(2), within the transmembrane region of the SERCA1a pump. The analysis employed the Molecular Operating Environment (MOE) software to calculate physicochemical properties, docking scores, and interaction energies.

**Fig. 5:**
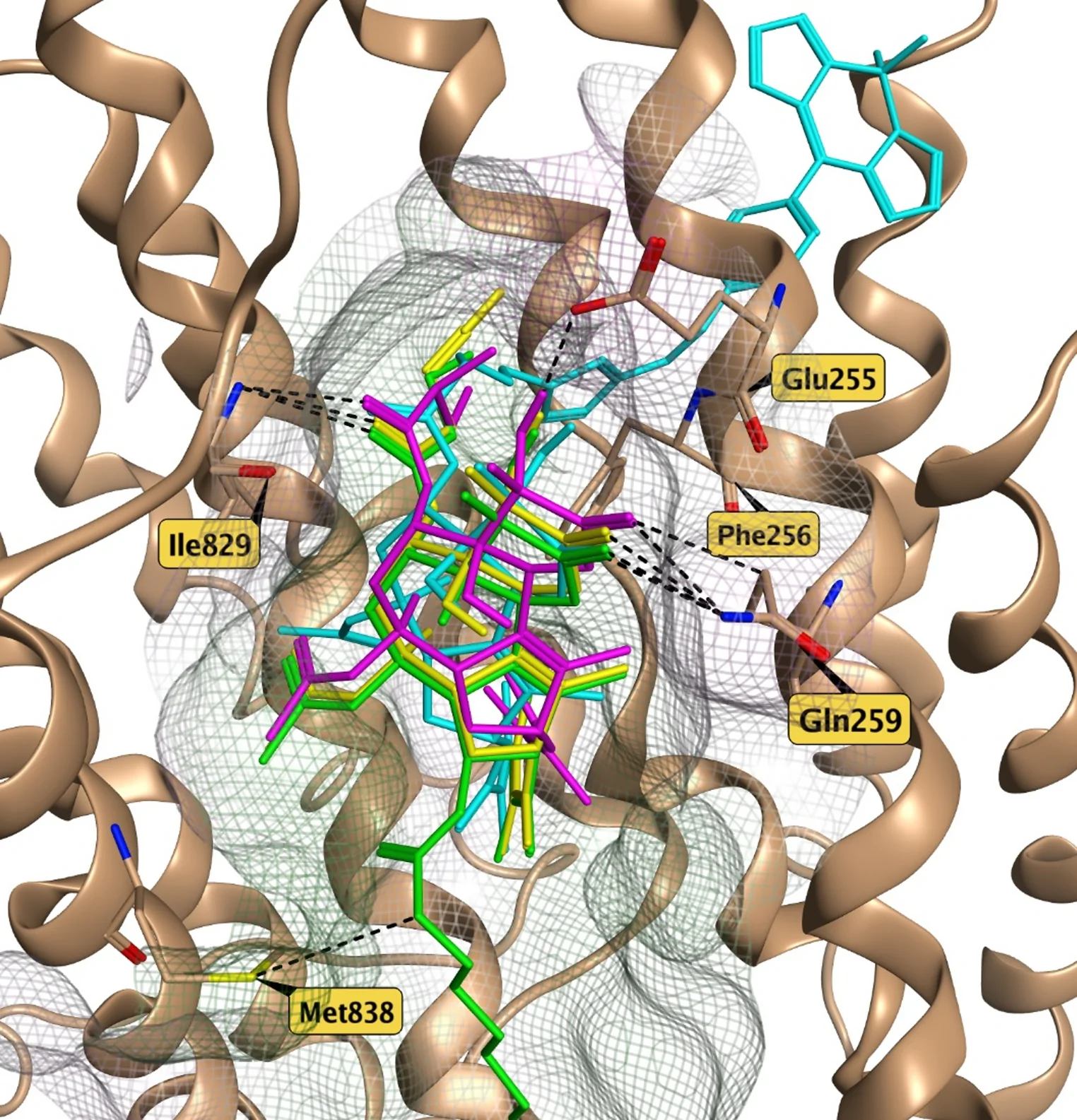
Binding poses of SERCA1a tested compounds within the transmembrane binding pocket (E2 conformation, PDB 2ear). Ligands thapsigargin (TG, green), trilobolide (TB, magenta), TB-44 (cyan), and TB-46(2) (yellow) are shown in stick representation within the SERCA1a transmembrane binding pocket (gray mesh). Key binding site residues—Glu255, Phe256, Gln259, Ile829, and Met838—are highlighted in atom color style. All four compounds adopt overlapping binding orientations anchored by conserved interactions at Phe256 (hydrophobic) and Gln259 (hydrogen bond), indicating a shared pharmacophore. See Table 4 for detailed residue-specific interactions

The binding sites of all four compounds were localized within the transmembrane region of SERCA1a, with TG positioning derived directly from the X-ray crystal structure serving as a reference ([33–35], pdb 2ear) (Fig. 5).

A central feature of sesquiterpene lactones and their derivatives recognition involves extensive hydrophobic contacts with Phe256, which forms a conserved interaction interface for TG, TB-44, TB, and TB-46(2) (Fig. 5). This aromatic residue acts an anchor point, thereby enhancing the energetic favorability of ligand binding through π-π stacking and van der Waals interactions. The consistency of Phe256, Gln259, and Ile829 engagement across studied compounds suggests that they are critical determinants of the SERCA1a inhibitor pharmacophore.

Computational analysis of ligand energetics revealed distinct thermodynamic and physicochemical profiles for each compound. The lipophilicity of the tested compounds, assessed through distribution and partition coefficients (logD₇.₄ and logP), ranged from highly hydrophobic for TB-44 (logP = 5.42) to relatively polar for TB-46(2) (logP = 0.26). Computational analysis revealed that TB-44 exhibited the strongest electrostatic contribution (ΔE_elst = -46.1 kJ/mol) and highest total interaction energy (ΔE = -122.9 kJ/mol), while TB showed a more balanced electrostatic-hydrophobic profile (ΔEelst = -36.6 kJ/mol; ΔE = -87.1 kJ/mol) (Table 3).

The GBVI/WSA ΔG binding scores, which incorporate implicit solvation effects through the Generalized Born Volume Integration/Weighted Surface Area model, represent predicted binding affinity of the tested compounds to SERCA1a – distinct from the experimental ΔΔG values (Tables 1 and 2) that measure changes in substrate binding affinity. GBVI/WSA ΔG scores ranged from − 8.8 kcal/mol for TB-46(2) to − 11.4 kcal/mol for TB-44, with TB-44 demonstrating the most favorable docking score and stronger predicted binding interactions than the gold-standard inhibitor TG (− 10.2 kcal/mol). However, these computational predictions do not directly correlate with experimental potency – TB paradoxically shows a higher potency (IC₅₀ = 0.36 µM) despite an intermediate GBVI/WSA ΔG (− 9.1 kcal/mol), indicating that static docking scores alone are insufficient to predict functional inhibitory potency. These scores instead align with the understanding that favorable static binding is achieved through optimization of both shape complementarity and energetic contributions from hydrophobic interactions and specific polar contacts. 

**Table 3:** Physicochemical properties and computational interaction energies of thapsigargin (TG), trilobolide (TB), and their analogs

	logD_7.4_	logP	GBVI/WSA ΔG score	ΔE (kJ/mol)	ΔE_elst_ (kJ/mol)	ΔE_vdw_ (kJ/mol)
TG	5.01	5.01	-10.2	-89.7	-26.8	-63
TB	2.43	2.43	-9.1	-87.1	-36.6	-50.4
TB-44	5.42	5.42	-11.4	-122.9	-46.1	-76.8
TB-46-2	0.78	0.26	-8.8	-84.2	-29.9	-54.3

Calculations were performed using MOE (version 2019.01). The GBVI/WSA ΔG values represent predicted binding free energies of each ligand to SERCA1a (kcal/mol) from molecular docking, calculated after induced-fit refinement using the MOE GBVI/WSA scoring function. The ΔE terms correspond to the total interaction energy, with the electrostatic component (ΔE_elst_) and van der Waals component (ΔE_vdw_), both expressed in kilojoules per mole (kJ/mol)

Detailed analysis of protein-ligand contacts revealed both conserved and compound-specific interaction patterns. All four compounds interacted with Gln259 via hydrogen bonds, establishing this residue as essential for maintaining ligand orientation within the binding cavity. Ile829 formed hydrogen bonds with TG, TB, and TB-44, but not with TB-46(2), reflecting the impact of chemical modifications on interaction specificity. TB-44 engaged Glu255 via hydrogen bonding, a unique contact not observed with other compounds (Table 4). In contrast, TG uniquely binds to Met838, whereas TB achieves high potency (IC₅₀ = 0.36 µM) without engaging either Glu255 or Met838, suggesting that multiple alternative binding modes can achieve effective SERCA1a inhibition.

**Table 4:** Predicted residue-specific interactions between SERCA1a (E2 conformation, PDB 2ear) and sesquiterpene lactones based on molecular docking in MOE

	Glu255	Phe256	Gln259	Ile829	Met838
TG		Hydrophobic	H-bond	H-bond	H-bond
TB		Hydrophobic	H-bond	H-bond	
TB-44	H-bond	Hydrophobic	H-bond	H-bond	
TB-46-2		Hydrophobic	H-bond	H-bond	

Hydrogen bonds (H-bond) and hydrophobic interactions were identified within 5 Å of the ligand binding pocket defined around thapsigargin’s crystallographic site. All tested compounds interact with Gln259 and Phe256, whereas TB-44 uniquely forms a hydrogen bond with Glu255, suggesting enhanced stabilization within the transmembrane cleft

## Discussion

The present study demonstrates that trilobolide (TB) and its synthetic derivatives TB-44 and TB-46(2) inhibit SERCA1a through a mechanism fundamentally similar to thapsigargin (TG), yet with notable differences in potency. Under optimal assay conditions, TB emerged as the most potent inhibitor (IC₅₀ = 0.36 ± 0.06 µM), with greater inhibitory potency than TG (IC₅₀ = 0.6 ± 0.12 µM). The structural modifications introduced in TB-44 and TB-46(2) resulted in substantial reductions in inhibitory potency, with IC₅₀ values of 1.47 ± 0.4 µM and 3.47 ± 1.8 µM, respectively. This approximately 4- to 10-fold attenuation relative to the parent compound, TB, provides valuable insights into the structural determinants of sesquiterpene lactone recognition by SERCA1a.

### Impact on ATP Binding and Catalytic Efficiency

Kinetic analysis of ATP-dependent SERCA1a activity reveals that all four tested compounds reduce both substrate binding affinity (increased K_m_) and maximal turnover rate (decreased V_max_). The magnitude of these changes varies across the compound series: TG produced the most dramatic V_max_ reduction (3.7% of control) with a modest K_m_ increase, whereas TB-44 and TB-46(2) showed more moderate effects on both parameters. However, TB’s higher potency (IC₅₀ = 0.36 µM) despite substantial ATP-affinity loss (ΔpATP₅₀ = −0.97) indicates that ATP-site disruption alone does not determine potency. These results are consistent with inhibitory mechanisms that impair multiple catalytic steps, as documented for TG-class compounds in prior structural studies [36–38].

### Differential Effects on Calcium-Dependent Activation

Sesquiterpene lactones and their derivatives bind universally, reducing V_max_ (21–72% of control) while preserving Ca²⁺ sensitivity (minimal K₀.₅ changes). This dissociation between impaired catalytic capacity and maintained Ca²⁺ recognition is consistent with inhibitory mechanisms that disrupt ATP-driven conformational cycling rather than directly disrupting divalent-cation binding. Notably, pCa₅₀ analysis reveals divergent profiles: TG, TB-44, and TB-46(2) show enhanced apparent Ca²⁺ affinity (negative ΔΔG), while TB exhibits reduced affinity (positive ΔΔG). This heterogeneity suggests that the inactive conformations stabilized by different sesquiterpene lactones may differ subtly in how they present the Ca²⁺-binding pocket. These findings apply uniformly to both natural inhibitors (TG, TB) and TB-derived probes (TB-44, TB-462), indicating that probe modifications preserve the core SERCA1a inhibitory mechanism [36–38].

### Lipophilicity and Structure-Activity Insights

Molecular docking revealed overlapping binding sites for all four compounds within the transmembrane region, engaging conserved residues including Phe256, Gln259, and Ile829. The universal engagement of Phe256 through hydrophobic interactions confirms its role as a critical pharmacophore anchor – its mutation significantly decreases thapsigargin sensitivity. The crystal structure of SERCA1a identified transmembrane helices M3, M5, and M7 involved in thapsigargin binding [36, 37], and hydrophobic interactions with these residues dominate ligand recognition in the E2 conformation.

These findings are consistent with experimental and computational studies showing that hydrophobic interactions with conserved pocket residues dominate ligand binding in the E2 conformation [37] and can accommodate certain structural variations.

Sesquiterpene lactone potency is modulated by both structural features and physicochemical properties. Classical structure-activity relationship studies of sesquiterpene lactones have established that moderate lipophilicity enhances both membrane permeability and binding to the hydrophobic transmembrane pocket [38, 39]. Trilobolide shares a similar sesquiterpene lactone scaffold with TG, including acetoxy or acyl groups at corresponding positions (*O*-3, C-4, *O*-8, *O*-10) that provide key hydrophobic interactions [36, 40]. It was discovered that membrane lipids play an important role not only in the binding of TG but also in modulating the affinity of TG at the binding site [34]. Structural modifications on trilobolide, replacing acetyl groups with bulkier moieties (BODIPY in TB-44) or more polar groups (azido in TB-46(2)), alter both lipophilicity and steric complementarity within the narrow transmembrane cleft. This results in changed interactions with peripheral residues: TB-44 uniquely engages Glu255 (hydrogen bond), while TB-46(2) loses these key peripheral contacts, suggesting that both core and peripheral interactions contribute to potency.

TB’s higher experimental potency (IC₅₀ = 0.36 µM) despite its intermediate computational binding energy (GBVI/WSA ΔG = − 9.1 kcal/mol, ΔE = − 87.1 kJ/mol) suggests that its moderate lipophilicity (logP = 2.43) strikes an optimal balance between membrane partitioning and productive engagement of the binding pocket. In contrast, TB-44’s high lipophilicity (logP = 5.42) produces stronger overall binding energy (− 11.4 kcal/mol) but weaker potency (IC₅₀ = 1.47 µM), suggesting that excessive hydrophobicity may impair aqueous solubility or kinetic accessibility. TB-46(2)’s low lipophilicity (logP = 0.26) and weakest potency (IC₅₀ = 3.47 µM) indicate that insufficient membrane partitioning limits effective concentration at the binding pocket.

These results indicate that optimal inhibition requires balancing membrane permeability, binding pocket fit, and peripheral interactions.

### TB-44 and TB-46(2) as Chemical Biology Probes

While TB-44 and TB-46(2) show reduced potency compared to TB, their rational design features position them as valuable chemical biology probes. TB-44’s BODIPY fluorophore enables real-time visualization of SERCA1a engagement in live cells and tissues using fluorescence microscopy, facilitating mechanistic studies of inhibitor kinetics and subcellular localization (21). TB-46(2)’s azido handle supports bioorthogonal labeling via copper-catalyzed click chemistry and activity-based protein profiling, enabling unbiased identification of off-target SERCA1a interactions and cellular targets. Beyond individual applications, these probes can serve as leads for compound library screening to discover novel SERCA1a modulators with improved pharmacological properties. The moderate-to-weak potency of these derivatives, combined with their unique functional capabilities, makes them ideally suited for biological interrogation rather than therapeutic development, a distinction from the parent inhibitors (TB and TG).

### Potential Physiological Implications of SERCA1a Inhibition

SERCA1a inhibition has been recognized an efficient approach to deplete ER Ca²⁺ stores, activate the unfolded protein response (UPR), and induce ER stress, ultimately leading to caspase-mediated apoptosis. This mechanism has been proposed as a potential therapeutic strategy for cancer treatment [41]. Thapsigargin is the best-characterized SERCA1a inhibitor and serves as an important reference point: it blocks SERCA1a with sub- to low-nanomolar potency, selectively over plasma membrane Ca²⁺-ATPase and Na⁺,K⁺-ATPase, by binding to the Ca²⁺-free E2 conformation and forming an essentially irreversible inhibitory complex [42]. In multiple tumor models, TG-induced ER Ca²⁺ depletion triggers UPR pathways (PERK–ATF4–CHOP, IRE1–XBP1, ATF6) up‑regulates DR5, and elicits caspase‑8–dependent apoptosis and JNK/MAPK activation in cancer cells, thereby functionally “sensitizing” tumor cells to death-receptor-mediated killing, including TRAIL-based approaches (reviewed by Jaskulska et al. [43]). Thus, our findings on SERCA1a inhibition should be interpreted within this well-established framework, whereby alterations in SR/ER Ca²⁺ content critically shape cellular Ca²⁺ homeostasis, ER stress signaling, and ultimately cell fate.

A major obstacle to the direct clinical use of TG as an antineoplastic agent is its lack of tumor selectivity. Therefore, the development of TG-based prodrug strategies, in which the active compound is released selectively at the tumor site via protease-cleavable peptide linkers or targeted delivery carriers, represents a promising therapeutic approach.

### Summary

This present study demonstrates that inhibitory potency among thapsigargin-class sesquiterpene lactones is determined by a combination of lipophilicity, membrane partitioning, and electrostatic–hydrophobic complementarity at the transmembrane binding site, rather than by binding affinity alone. Trilobolide (TB; IC₅₀ = 0.36 µM) exceeds thapsigargin (TG; IC₅₀ = 0.60 µM) in potency despite a lower predicted binding score, an outcome explained by its moderate lipophilicity (logP 2.43) and stronger electrostatic contacts with Gln259. Across all four compounds, inhibition is expressed predominantly as suppression of maximal catalytic turnover (V_max_) with preservation of Ca²⁺ sensitivity. This kinetic profile—reduced catalytic capacity coupled with maintained Ca²⁺ recognition—is consistent with mechanisms proposed for thapsigargin-class inhibitors, which are known to stabilize inactive SERCA1a conformations and impair ATP-driven catalysis.

Structural modifications in TB-44 (BODIPY) and TB-46(2) (azide) were associated with four- to tenfold reductions in potency relative to TB. Docking analysis revealed that TB-46(2) additionally lost the hydrogen bond contact with Ile829 present in TG, TB, and TB-44, suggesting that both physicochemical and geometric factors contribute to attenuated inhibitory efficacy. Given the functional groups introduced into their scaffolds, TB-44 and TB-46(2) are regarded as chemical biology probes rather than drug candidates: TB-44 enables fluorescence-based tracking of SERCA1a engagement in intact cells, and the azide handle of TB-46(2) supports bioorthogonal labeling and activity-based protein profiling. These attributes position TB-44 and TB-46(2) as a structurally defined probe set for mechanistic investigation of SERCA1a function and for screening compound libraries targeting ER/SR Ca²⁺ homeostasis.

## Data Availability

Data will be made available on request.
